# Time-Point Dependent Activation of Autophagy and the UPS in SOD1G93A Mice Skeletal Muscle

**DOI:** 10.1371/journal.pone.0134830

**Published:** 2015-08-05

**Authors:** Sara Oliván, Ana Cristina Calvo, Samanta Gasco, María Jesús Muñoz, Pilar Zaragoza, Rosario Osta

**Affiliations:** Laboratorio de Genética y Bioquímica (LAGENBIO), Facultad de Veterinaria, Instituto Agroalimentario de Aragón (IA2), Instituto de Investigación Sanitaria Aragón, Universidad de Zaragoza, Zaragoza, Spain; University of Rome La Sapienza, ITALY

## Abstract

Amyotrophic Lateral Sclerosis (ALS) is a fatal neurodegenerative disease characterized by a selective loss of motor neurons together with a progressive muscle weakness. Albeit the pathophysiological mechanisms of the disease remain unknown, growing evidence suggests that skeletal muscle can be a target of ALS toxicity. In particular, the two main intracellular degradation mechanisms, autophagy and the ubiquitin-proteasome degradative system (UPS) have been poorly studied in this tissue. In this study we investigated the activation of autophagy and the UPS as well as apoptosis in the skeletal muscle from SOD1G93A mice along disease progression. Our results showed a significant upregulation of proteasome activity at early symptomatic stage, while the autophagy activation was found at presymptomatic and terminal stages. The mRNA upregulated levels of *LC3*, *p62*, *Beclin1*, *Atg5* and *E2f1* were only observed at symptomatic and terminal stages, which reinforced the time-point activation of autophagy. Furthermore, no apoptosis activation was observed along disease progression. The combined data provided clear evidence for the first time that there is a time-point dependent activation of autophagy and UPS in the skeletal muscle from SOD1G93A mice.

## Introduction

Autophagy and the ubiquitin-proteasome degradative system (UPS) are considered two main critical mechanisms that contribute to basal elimination of misfolded proteins and hence, they maintain the balancing protein homeostasis inside cells. Albeit autophagy was initially described as a central mechanism for cell survival under starvation conditions, a growing number of studies indicate that autophagy can play a relevant role in eliminating protein aggregates, especially in neurodegenerative diseases, mainly because its activation can efficiently degrade misfolded mutant proteins[1].

ALS is one of the most known neurodegenerative diseases characterized by a selective loss of motor neurons together with a progressive muscle weakness. The first studies that connected an impairment in autophagy to the progression of ALS showed a significant upregulation of the activated microtubule-associated protein 1 light chain (LC3) protein levels at the end symptomatic stage in the spinal cord from SOD1G93A mice, one of the best characterized animal models of the disease[2, 3]. At this step, autophagy, an intracellular process that leads to the elimination of cytoplasmic components inside lysosomes, was proposed as the main process that degraded mutant SOD1 *in vitro* and *in vivo* in the spinal cord from SOD1G93A mice[4]. Remarkably, a wide range of studies have characterized till now the possible cause of autophagy impairment in different experimental models for ALS[5–13]. In addition, mutant SOD1 aggregates not only induce impairment in proteasome activity but also alterations in SOD1 intracellular distribution[14]. In particular, there is clear evidence that endoplasmic reticulum (ER) stress correlates directly to motor neuron loss and ALS progression and interestingly, the adaptative responses to ER stress through protein folding pathways, such as unfolded protein response (UPR) are also closely related to autophagy in mutant SOD1 transgenic mice [7, 8]. The UPR is the main protective mechanism during ER stress and it promotes either protein folding or degradation of misfolded proteins. Precisely, the UPS is an important mechanism for the degradation of misfolded proteins exporting from ER and its failure can lead to significant ER stress[7, 15–17].

In this complex scenario and taking into consideration that the main cause of ALS disease remains unknown, the need for studying autophagy not as a single process but just in connection with other related processes such as the UPS, becomes relevant to a better understanding of the molecular basis of ALS. It is worth noting that both autophagy and proteasome were proposed as essential mechanisms for the reduction of mutant SOD1-mediated neurotoxicity in familial ALS (fALS), the genetic form of the disease [5, 18]. However, little information has been reported regarding autophagy and proteasome activity in one of the most affected tissues of the disease, the skeletal muscle, which is a more accessible tissue than spinal cord or brain in potential therapeutic strategies. Actually, muscle weakness has been described as one of the major causes of disability in ALS and for this reason the better understanding of the way ALS can affect skeletal muscle function becomes essential.

As a matter of fact, previous studies have characterized the skeletal muscle as a primary target of mutant SOD1 toxicity and the need for modulation of autophagy as a potential therapeutic intervention to counteract muscle atrophy has been also proposed [9, 19, 20]. Recent data reported that mutant SOD1 clearance was higher in muscles than in motorneurons. Taking into consideration that proteasome impairment has been shown to trigger autophagy, in this study, we investigated the role of autophagy in skeletal muscle tissue from SOD1G93A mice along disease progression and in connection with the UPS. Our results revealed a novel finding about the time-point dependent activation of autophagy and the UPS in this tissue.

## Materials and Methods

### Animals and skeletal muscle samples

Transgenic mice expressing the G93A mutant human SOD1 were originally obtained from The Jackson Laboratory (B6SJLTg(SOD1*G93A)1Gur/J). Hemizygotic transgenic mice were obtained by mating transgenic males with F1 females (B6SJL). The offspring were identified by PCR amplification of DNA extracted from tail tissue with specific primers for human SOD1. Wild type (WT) littermates were used as controls for all experiments. Hemizygous SOD1G93A mice at the following days of life were used: 40 (presymptomatic, P40), 60 (early symptomatic, P60), 90 (symptomatic, P90) and 120 (terminal stage, P120). The animals were housed in the Unidad Mixta de Investigación of the University of Zaragoza under a 12h light/dark cycle. Food and water were available ad libitum. The care and use of animals were performed accordingly with the Spanish Policy for Animal Protection RD53/2013, which meets the European Union Directive 2010/63/UE on the protection of animals used for experimental and other scientific purposes. All of the experimental procedures were approved by the in-house Ethic Committees for Animal Experiments of the University of Zaragoza.

All experimental groups were balanced to avoid sex bias [21]. The mice were sacrificed by CO_2_ anesthesia and skeletal muscles from both hindlimbs were dissected. Skeletal muscle tissues, for gene and protein expression studies, were snap-frozen and preserved at -80°C until to be pulverized in liquid nitrogen with a cell crusher. The powdered tissue was divided equally for RNA and protein extraction. For TUNEL and immunofluorescence assays, skeletal muscle tissue was removed, fixed with 4% paraformaldehyde in phosphate-buffered saline (PBS) for 24 hours and cryopreserved in 30% sucrose for 48 hours. Sample tissues were embedded in OCT (Tissue-Tek, Sakura Finetek) and frozen in liquid nitrogen-cooled 2-methylbutane. The tissue blocks were stored at -80°C.

### mRNA expression

For RNA extraction, powdered muscle tissue was homogenized with Trizol Reagent (Invitrogen) (n = 10 animals per time point and genotype, 5 males and 5 females) using a PRO200 homogenizer (PRO Scientific Inc.). RNA extracted was treated to eliminate genomic DNA using the Turbo DNA-free kit (Ambion). Reverse transcription was carried out according to the SuperScript First-Strand Synthesis System kit (Invitrogen). Gene expression variations in all of the samples were assayed by real-time PCR in an ABI Prism 7000 Sequence Detection System (Applied Biosystems). Primer and probe mixtures for each gene (*LC3* (Mm00458724_m1), *p62* (Mm00448091_m1), *Beclin1* (Mm00517174_m1), *Atg5* (Mm00504340_m1) and *E2f1* (Mm00432939_m1)) were supplied by Applied Biosystems. Two endogenous genes (*GAPDH* and *β-actin*) were used for normalization of the data [22, 23]. All reactions were performed in triplicate and the reaction efficiencies of the primer/probe sets were close to 100%.

### Immunoblot analysis

The powdered skeletal muscle tissue (n = 12 animals per time point and genotype, 6 males and 6 females) was resuspended in RIPA lysis buffer with protease inhibitors (Complete, Roche) and the homogenate were centrifuged at 10.000 g for 10 min at 4°C. The supernatants were collected and the protein concentration was quantified by BCA method (Sigma-Aldrich). Forty micrograms of protein was resolved on a 15% SDS-page gel and the proteins were transferred to PVDF membranes (Amersham Biosciences). The membranes were blocked in Tris-buffered saline supplemented with 0.1% Tween 20 and 5% (w/v) powered skim milk overnight at 4°C and then incubated with primary antibodies for one hour at room temperature (RT) against LC3 (1:1000; PD014, MBL), p62/SQSTM1 (1:1000; BML-PW9860, Enzo Life Sciences), Beclin1 (1:500; sc-11427, Santa Cruz), Caspase-3 (1:500; 9662, Cell Signalling), Caspase-9 (1:500; 9508, Cell Signalling), Bax (1:1000; sc-526, Santa Cruz), Bcl2 (1:1000; sc-492, Santa Cruz), PARP-1 (1:500; sc-7150, Santa Cruz) and GAPDH (1:1000; sc-25778, Santa Cruz). After incubation with HRP-conjugated secondary antibodies, bands were visualized by ECL reagents (GE Healthcare Life Science). Inmunoblots were scanned and densitometry was measured with AlphaEase FC software (Bonsai). Results were normalized to the corresponding GAPDH signal.

### Inmunofluorescence

Transverse sections of skeletal muscle (10 μm) were cut with using a cryostat (CM1510S Leica Microsystems). Tissue sections were re-fixed on ice with formalin solution 10% (HT5014, Sigma) for 10 min, permeabilized with 0.1% Triton X-100 and 0.1% sodium citrate in PBS for 10 min at RT, and blocked with 10% goat serum and 1% BSA in PBS for 30 min at RT. After washes, sections were incubated with primary antibodies over night at 4°C against Beclin1 (1:50; sc-11427, Santa Cruz) and LC3 (1:200; PD014, MBL), and subsequently incubated with Alexa Fluor 546 goat anti-rabbit IgG (Invitrogen) for 1 h at RT. Nuclear staining (in blue) was performed using a mounting medium with DAPI (Vectashield, H-1200, Vector Laboratories) and visualized on an Olympus IX81 fluorescence microscope.

### Proteasome activity

Skeletal muscle tissue was homogenized in cold buffer (20 mM TrisHCl pH 7.5, 2 mM EDTA) and centrifuged at 15000 g for 10 min at 4°C (n = 6 animals per time point and genotype, 3 males and 3 females). Protein concentration in supernatants was determined using the BCA protein assay. All samples were assayed in triplicate using 10 μg of freshly protein extracts. Proteasome activity was measured using the CHEMICON Proteasome Activity Assay Kit (APT280, Millipore) as described by the manufacturer. The extracts were incubated (2 h at 37°C) with a labelled substrate, LLVY-7-amino-4-methylcoumarin, and the cleavage activity was monitored by detection of the free fluorophore 7-amino-4-methylcoumarin using a fluorescence plate reader (Infinite F200 PRO, TECAN) at 360/460 nm.

### Chloroquine treatment

Skeletal muscle tissue (n = 6 SOD1G93A mice at P120, 3 males and 3 females) were dissected and the tissue was then chopped into small pieces. The explants were rinsed in phosphate-buffered saline (PBS) prior to being cultured in DMEM containing 10% (v/v) fetal bovine serum and 100 μM chloroquine (C6628,Sigma-Aldrich) for 6 hours at 37°C and 5% CO2. After culture, the explants were washed twice with PBS, snap-frozen and preserved at -80°C. For protein extraction, explants were processed as described above and LC3-II expression was assessed.

### Apoptotic detection

Detection of apoptotic nuclei was assessed by TUNEL assay (Terminal deoxynucleotidyl transferase fluorescein-dUTP nick end-labelling) using the In situ Cell Death Detection kit (Roche) in transverse sections of 10 μm. The positive controls were carried out on serial sections after prior incubation with DNAse I, while negative control used label solution instead of TUNEL reaction mixture. Slides were mounted with Vectashield with DAPI (Vectashield, H-1200, Vector Laboratories). The percentage of apoptotic cells was determined by counting TUNEL-positive cells on an Olympus IX81 fluorescence microscope.

### Statistical analysis

Results obtained from SOD1G93A and control groups were compared using Student’s *t*-test or ANOVA followed by Bonferroni post-hoc test. All of the values were expressed as means and error bars represent standard error of the mean (SEM). The statistical significance threshold was set at *p*<0.05. The software used for the statistical analysis was SPSS 19.0 (IBM).

## Results

### Activation of autophagy at presymptomatic and the late stages of the disease

**Fig 1:**
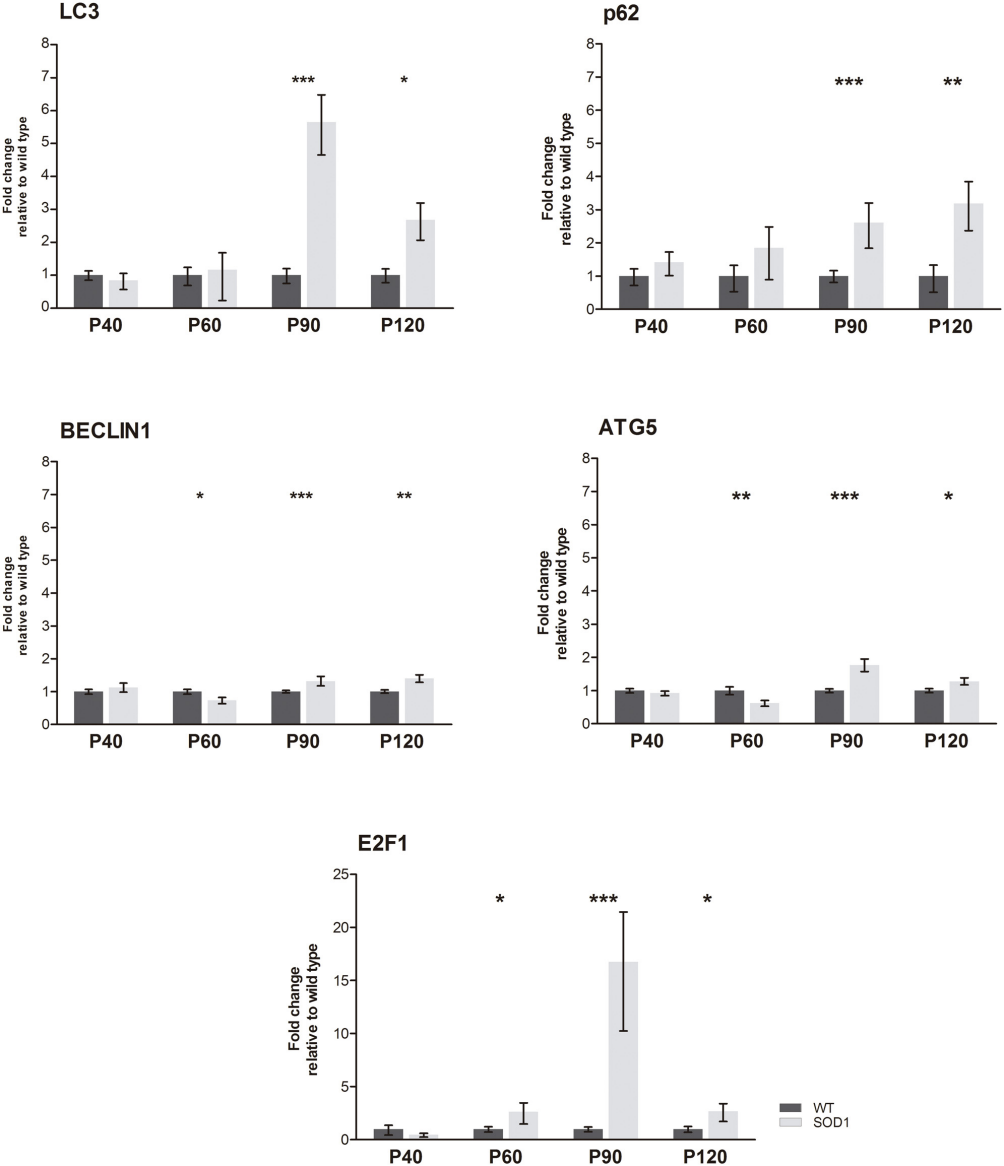
mRNA expression levels of autophagy markers. Relative expression values of *Lc3*, *p62*, *Beclin1*, *Atg5* and *E2f1* in SOD1G93A mice (grey bars) and wild type mice (WT, black bars) at each studied stage. Each data point represented the mean ± SEM. n = 10 animals per time-point and genotype. *p <0.05, **p <0.01 and ***p <0.001 versus age-matched WT.

To characterize the autophagy machinery in SOD1G93A mice along disease progression, the mRNA levels of *Lc3*, *p62*, *Beclin1*, *Atg5* and *E2f1* genes were first quantified by real-time PCR in the skeletal muscle tissue of WT and SOD1G93A mice at P40, P60, P90 and P120. At asymptomatic stage (P40) no significant changes were found. At early symptomatic stage (P60), mRNA levels of *Beclin1* and *Atg5* were significantly downregulated in SOD1G93A mice, while upregulated levels were found in the case of *E2f1*. However, at symptomatic and terminal stages of the disease (P90 and P120) all the analyzed transcripts were significantly upregulated (Fig 1).

**Fig 2:**
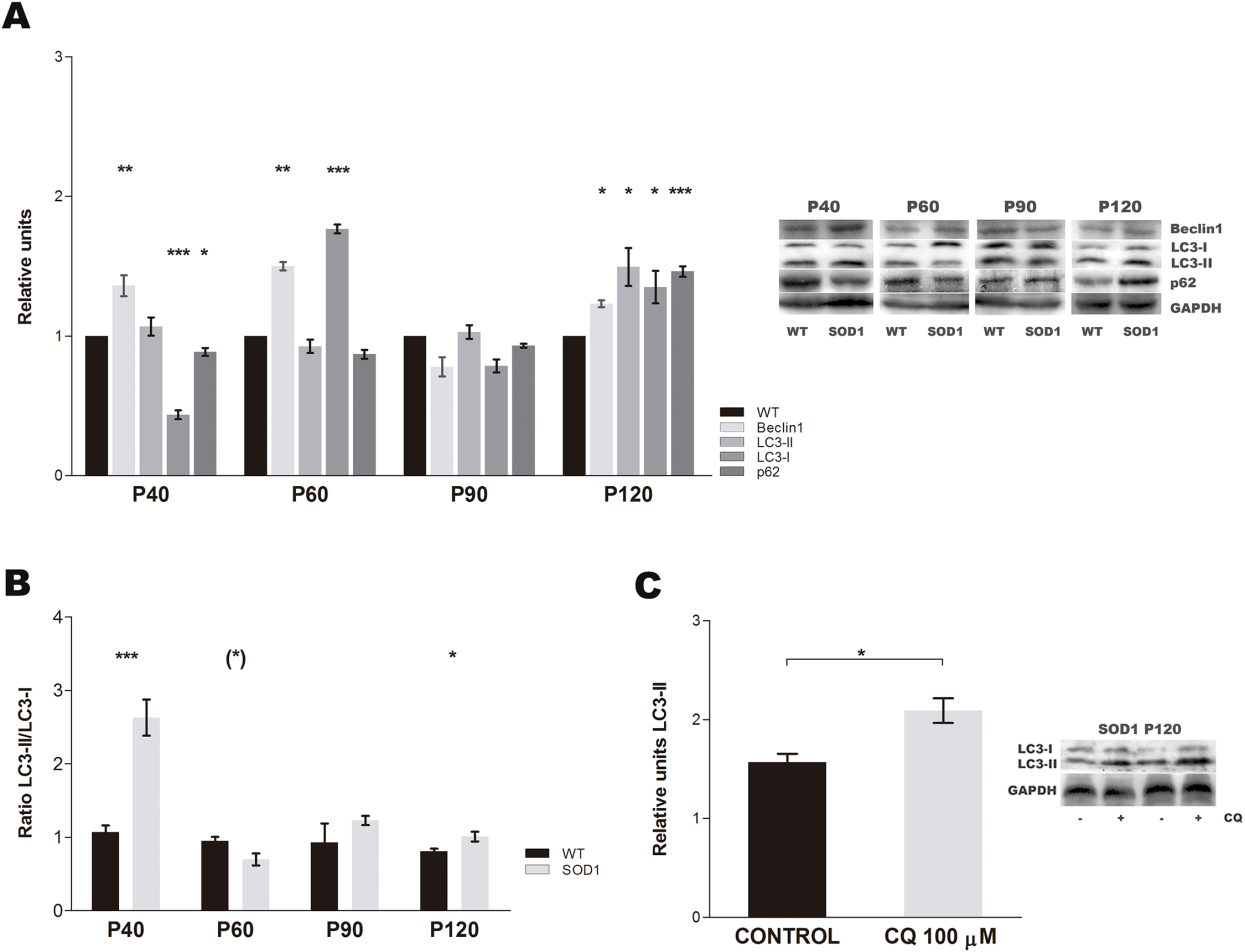
Autophagy protein expression. (A) Western blots for Beclin1, LC3-II, LC3-I and p62 protein levels in SOD1G93A mice (grey bars) and age-matched wild type mice (WT, black bars) along disease progression. The cropped blots portrayed are representative of independent experiments. All gels were run under exact same experimental conditions for best comparison. Data showed mean ± SEM, n = 12 animals per time-point and genotype. *p <0.05 and ***p <0.001 versus age-matched WT. (B) Results of LC3-II were normalised to the corresponding LC3-I signal to generate ratio LC3-II/LC3-I in SOD1G93A mice (light grey bars) and age-matched wild type mice (WT, black bars). Data showed mean ± SEM. *p <0.1, **p <0.01 and ***p <0.001. (C) Relative expression levels of LC3-II from SOD1G93A mice at P120, before (light grey bars) and after chloroquine treatment (dark grey bars). Data showed mean ± SEM. n = 6 animals. *p <0.05.

Moreover, we analyzed the protein expression of three widely used autophagy markers, Beclin1, LC3 and p62 along disease progression. The expression of Beclin1 was significant increased in all stages except at P90 when no differences were detected between WT and SOD1G93A. In case of LC3-II and p62, the expression levels of both proteins remained almost unchanged until P90. A statistically significant downregulation of p62 levels were observed at P40, while a significant impairment was found in LC3-I levels at the first stages of the disease. However, at P120, a significant upregulation was found in Beclin1, LC3-I, LC3-II and p62 (Fig 2A). Furthermore, the ratio LC3-II/LC3-I showed that the autophagy flux was significant increased at P40 and P120 while decreased at P60 (Fig 2B).

The upregulation of Beclin1, LC3-II and p62 levels at P120 could point out to an activation of autophagy process or just its blockage. Therefore, in order to shed light on this question, we next assessed the autophagic flux. At this point, skeletal muscle samples from SOD1G93A mice were treated with chloroquine, an agent that impairs lysosomal acidification. The results showed an accumulation of LC3-II, so this would indicate that the upregulated levels of Beclin1, LC3-II and p62 were a consequence of an enhancement of the autophagic flux (Fig 2C) [24].

**Fig 3:**
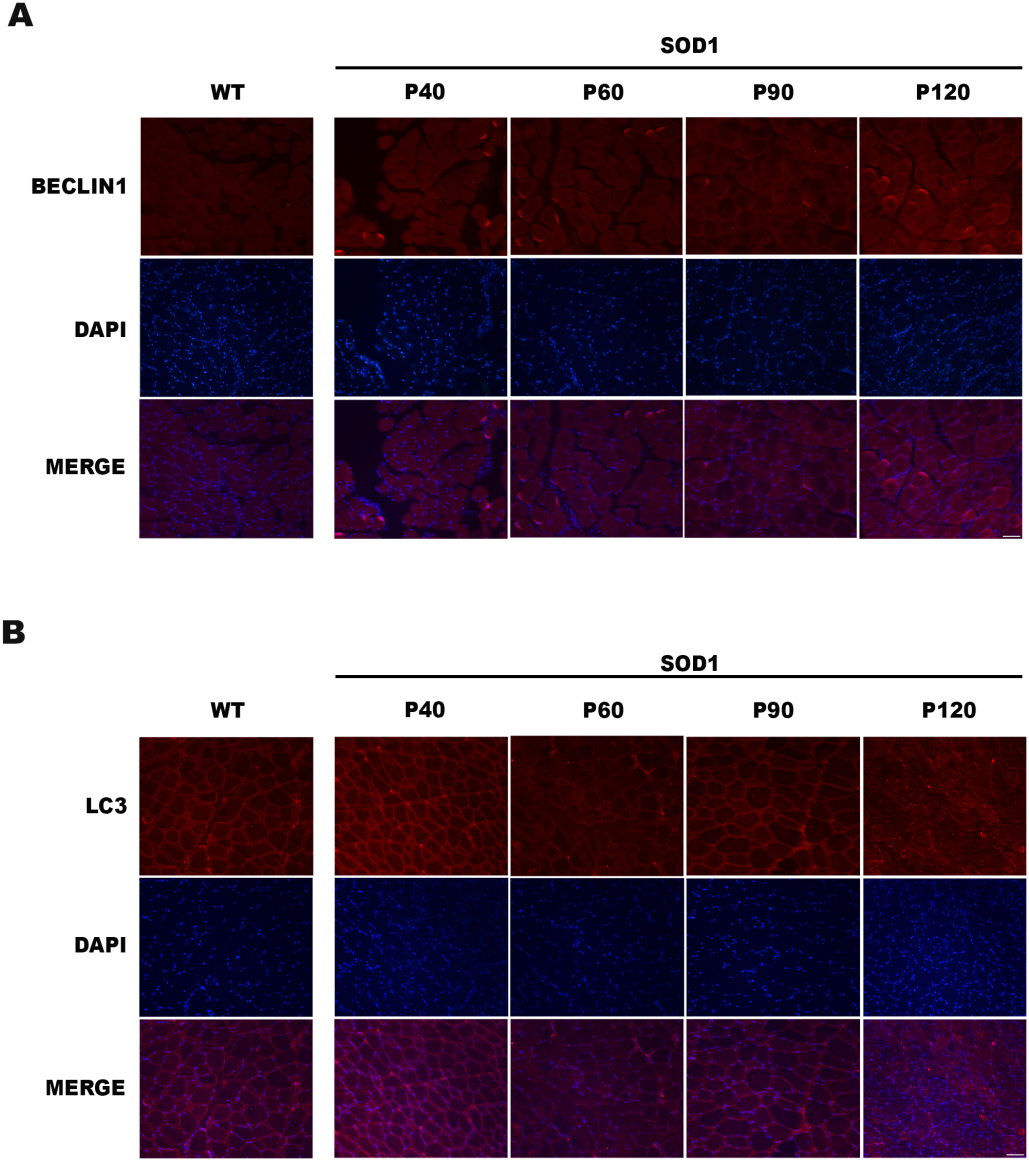
Immunofluorescence of Beclin1 and LC3 staining. Beclin1 (A) and LC3 (B) staining was performed in skeletal muscle tissue from wild type (WT) and SOD1G93A mice. DAPI staining was also performed (blue). A merged image of the double staining is presented. A representative image presents of 3 independent animals for genotype and disease stages. Scale bars: 100 μm.

Moreover, we also characterized the expression partner of Beclin1 and LC3 in the skeletal muscle tissue using immunofluorescence staining. The expression of both proteins was consistent with the pattern described above (Fig 3).

### Activation of proteasomal activity at early symptomatic stages

**Fig 4:**
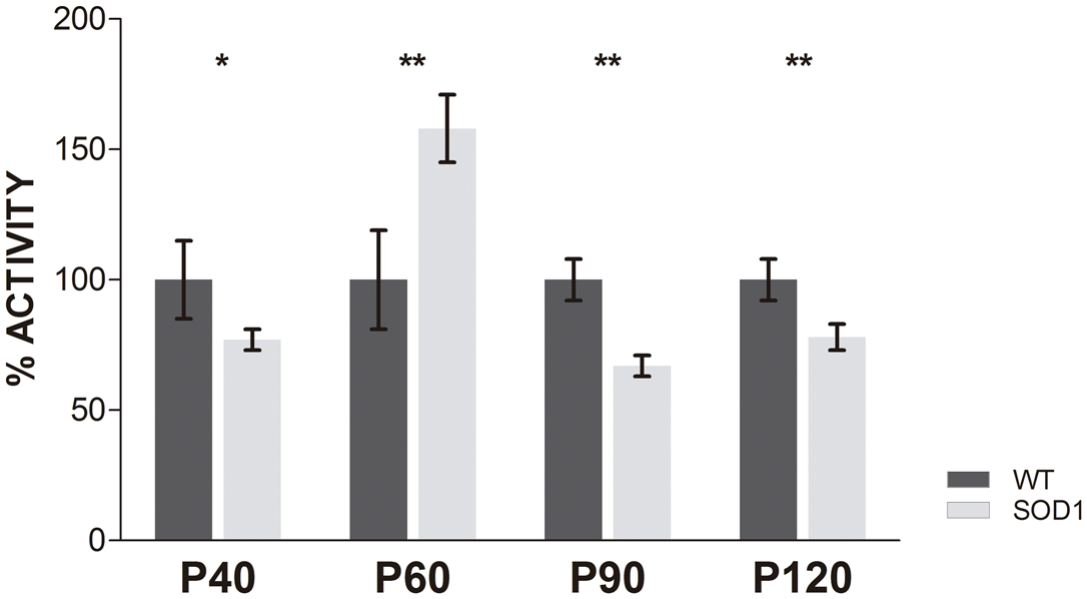
Proteasome activity. Proteasome activity was measured in skeletal muscle homogenates from SOD1G93A mice (grey bars) and age-matched wild type mice (WT, black bars) at each stage of the disease. Data showed mean ± SEM. n = 6 animals per time-point and genotype. *p <0.05 and ***p <0.001.

Proteasome activation is the primary pathway by which proteins are cleared from cells [25]. Given the late activation of autophagy in SOD1G93A mice, we further investigated whether autophagy impairment influenced proteasomal activation at early stages. For these purpose, we measured the activity of the 20S proteolytic component of the 26S proteasome. In SOD1G93A mice, the proteasomal activity was significantly reduced at P40, P90 and P120, while at P60 the proteasome was significantly activated (Fig 4). These results suggested an impairment in proteasome function in the skeletal muscle of SOD1G93A mice in the main stages of the disease and the activation peak at P60 could imply a time-dependent way of action in the proteasomal degradation.

### Absence of apoptosis activation in skeletal muscle tissue

**Fig 5:**
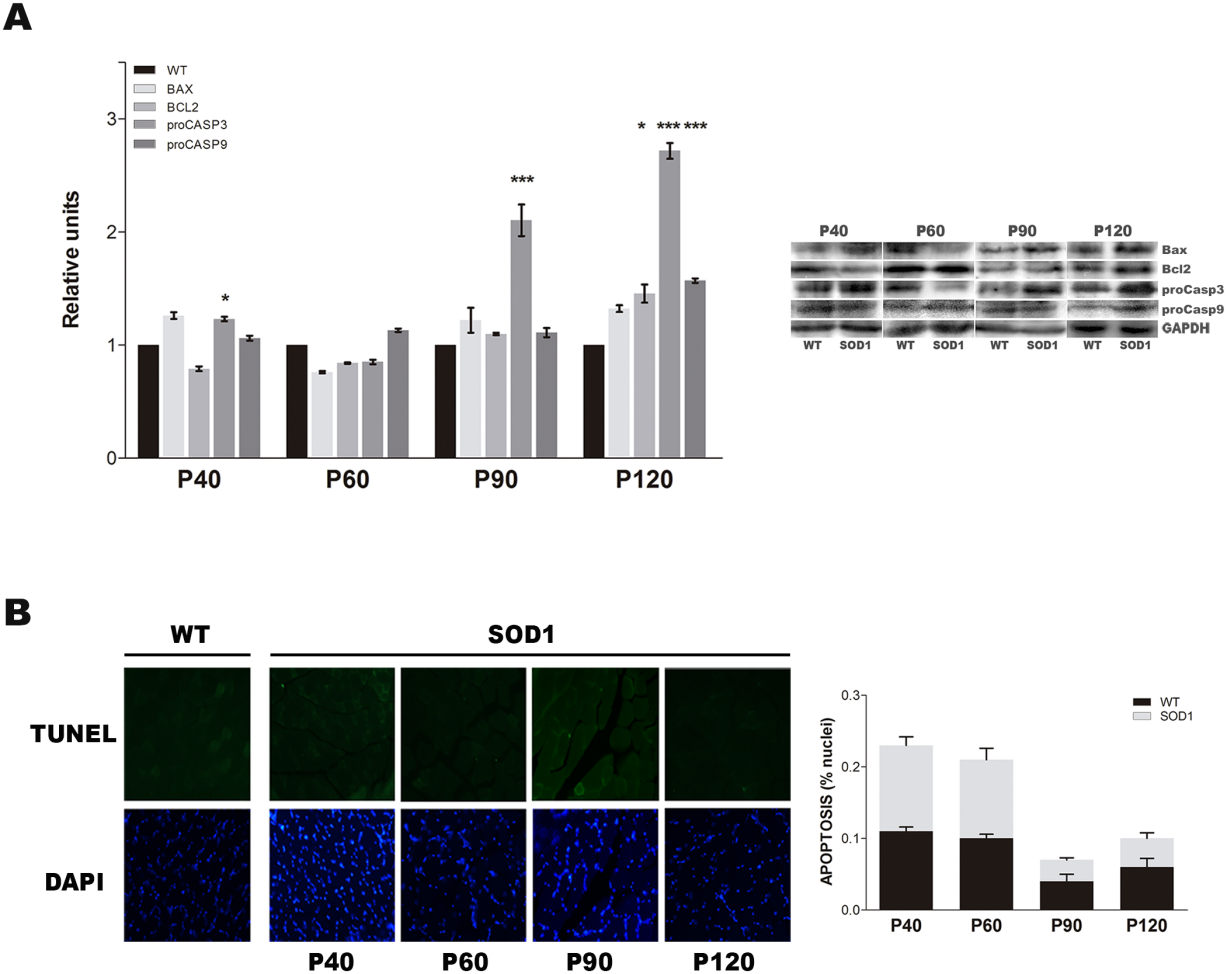
Apoptosis activity. (A) Protein expression profile of apoptotic markers, Bax, Bcl-2 and procaspase-3, in SOD1G93A mice (grey bars) and age-matched wild type mice (WT, black bars) along disease progression. Data showed mean ± SEM, n = 12 animals per time-point and genotype. *p <0.05 and ***p <0.001 versus age-matched WT. (B) Detection of apoptotic nuclei by TUNEL assay at each stage of the disease. Data showed mean ± SEM, n = 8 animals per time-point and genotype.

To further study in depth the role of the time-dependent activation of autophagy and proteasome in the disease, we determined the role of apoptosis in the progression of the disease. Western blot analysis for the total and the cleaved form of caspase-3, caspase-9, Bax, Bcl-2 and the poly ADP ribose polymerase (PARP) profile expression pattern was performed. The caspase-3 and caspase-9 has been described as the final effector of the apoptotic cell death mechanism meanwhile the proteolytic cleavage (activation) of PARP-1 has been considered as a hallmark biochemical feature of apoptosis,. Interestingly, the proteins caspase-3 and caspase-9 in its active form were no detected in WT or SOD1G93A mice as well as PARP-1 (S1 Fig). However, the highest procaspase-3 and procaspase-9 expression levels were found at P90 (*p*>0.001) and P120 (*p*>0.001) respectively. Moreover, the activity of Bcl-2, an anti-apoptotic protein, decreased significantly at P40 and increased significantly at the terminal stage, P120 (*p*<0.05) (Fig 5A). These data suggested that apoptosis was not exerting a decisive role in the skeletal muscle tissue from SOD1G93A mice. In addition, to verify the western blot results, the apoptosis was visualized by TUNEL assay. As shown in Fig 5B, the percentage of TUNEL positive cells was very low at any stage of the disease (less than 1%) in all the tested samples from WT or SOD1G93A mice. Moreover, not significant differences were detected between WT and SOD1G93A mice, supporting the fact that the apoptotic cell death was not happening in the skeletal muscle from SOD1G93A mice along disease progression.

## Discussion

Skeletal muscle is one of the target tissues dramatically affected by the degenerative progression of ALS. Actually, this tissue plays an important role in the neurophysiologic diagnosis of the disease and therefore it may be considered a primary target of ALS toxicity[26–28].

The role of autophagy in SOD1G93A mice was just described as one of the major intracellular mechanism involved in muscle atrophy[19]. The oxidative stress induced in this animal model is sufficient to activate autophagy. Interestingly, in MLC/SOD1G93A mice, in which the overexpression of mutated SOD1 is restricted to skeletal muscle, the siRNA inactivation of LC3, an essential marker for autophagosome formation, led to the rescue of the muscle phenotype[19]. Furthermore, not only autophagy but also the ubiquitin-proteasome degradative system (UPS) is more markedly induced in muscle cells than in motor neurons[29, 30].

In this study, we further investigated the activity of autophagy and the UPS as well as the apoptosis that could explain the induction of muscle atrophy in SOD1G93A mice.

In relation with autophagy, at presymptomatic stage (P40), the mRNA levels of *LC3*, *p62*, *Beclin1*, *Atg5* and *E2f1* in SOD1G93A mice were similar to WT mice. However, the expression of Beclin1 protein, a well-known key regulator of autophagy, and the ratio LC3-II/LC3-I was increased, so these results could be related with an autophagy activation. It has been described that the mutant SOD1G93A protein may promote the early increase in autophagic activity. Indeed, this activation has been observed both in skeletal muscle cell line C2C12 [9] and in the skeletal muscle fibers from SOD1G93A mice [31].

At the beginning of the disease (P60), *Beclin1* and *Atg5* levels significantly decreased meanwhile *E2f1* levels increased in SOD1G93A with respect to WT mice. Beclin1, Atg5 and E2f1 are components of the autophagy initiation complex; therefore, the *Beclin1* and *Atg5* deregulation suggested an autophagy inhibition in this time point in spite of the upregulated *E2f1* levels. In particular, Beclin1 plays a relevant role in the regulation and induction of a correct autophagy flux in muscle during fasting and precisely, a muscle specific inactivation of *Atg5* gene determines a severe muscle wasting in mice[32]. Therefore, the downregulation of these two markers pointed out to an insufficient autophagy, being detrimental for muscle homeostasis. Moreover, transcriptional activity of *E2f1* plays an important role in the induction of autophagy. Furthermore, it has been described that *E2f1* and its truncated form *E2f1tr*, lacking the transactivation domain, can upregulate the expression levels of autophagic genes such as *LC3* and *Atg5*[33, 34]. In spite of the well described role of E2f1 in the modulation of both apoptosis and autophagy, mainly in melanoma and embryonic cell lines, the upregulated levels of *E2f1* found in the skeletal muscle from SOD1G93A mice at P60 could not be sufficient enough to prompt the activation of *LC3* and, above all, *Beclin1* and *Atg5*. At protein level, the lower ratio LC3-II/LC3-I and an absence of increased LC3-II level suggested a decreased autophagosome formation and therefore a lack of autophagy activation in SOD1G93A mice. However, the expression of Beclin1 was increased. Beclin1 has been described to play a central role in autophagy and also in numerous biological processes including apoptosis [35]. In fact, it has been described an abnormal association of Beclin1 with mutant SOD1 in ALS disease that may alter the autophagy process [36].

Following with the autophagic response in skeletal muscle, at symptomatic stage P90, the mRNA expression of autophagy makers were upregulated, but at protein level, contrary to expectations, no differences were detected between WT and SOD1G93A mice. Thus, our data suggested an impairment in the autophagy of SOD1G93A mice. We hypothesized that at P90 the skeletal muscle could prompt a regenerative response instead of an autophagy activation. In fact, in the muscle from this animal model, the myogenic regulatory factors were mostly increased at protein level especially while at P120 no protein increase was observed [37].

Finally, at terminal stage, the increased expression both gene and protein level could be related with an autophagy activation. To determine if this upregulation was a consequence of a blockage or an activation of autophagy, we blocked the activity using chloroquine in SOD1G93A mice. Chloroquine inhibits autophagy by inhibiting the acidification of autophagosome and maturation of autolysosome, resulting in the accumulation of autophagic vesicles and the inhibition of autolysosomal protein degradation. The pharmacological inhibition of autophagy by chloroquine has been previously tested in *in vitro* models of ALS, but to date no data has been registered in skeletal muscle cells[38]. After treating with chloroquine the explants from skeletal muscles, significant LC3-II increased levels were observed, suggesting that autophagy was still activated. As a consequence, the upregulation of LC3-II and p62 indicated a significant activation of autophagy flux at this terminal stage. These data are consistent with previous studies in the same tissue, in which mRNA and protein levels of LC3 and p62 were found significantly increased in SOD1G93A and in MLC/SOD1G93A mice at later stages of the disease, suggesting that muscle cells were still able to upregulate these two essential markers of autophagy [19, 30].

The next question that arouse to us was the study of the mechanisms that could be in clear connection with autophagy to manage the degenerative progression of the disease in skeletal muscle tissue. The relation between autophagy and the UPS seems to be close enough to finely remove the intracellular accumulation of mutant SOD1 aggregates[15]. Actually, p62 participates in both mechanisms to eliminate ubiquitinated proteins from cells. At this step and taking into consideration the significant autophagy activation at P120, we wonder if the proteasome activation could start earlier than autophagy in the skeletal muscle. Previous studies in this animal model showed that soluble mutant SOD1 in skeletal muscle did not have direct effects on mitochondrial dysfunction, protein misfolding and muscle loss[30, 39]. However, the expression of mutant SOD1 in this tissue induced atrophy and mitochondrial abnormalities, favouring proteasome degradation under proteotoxic conditions, such as misfolded mutant SOD1 overexpression[30, 40]. In accordance with these studies, our results showed an impairment in proteasome activity along disease progression. In particular, a significant upregulation of proteasome activity was only found at P60when autophagy process was clearly inactivated. These results suggested that the proteasome degradation was only activated at early symptomatic stage in skeletal muscle tissue.

On the other hand, many works suggest that there is an interplay between apoptosis and autophagy [35, 41, 42]. In this interconnection, Beclin1 plays a critical role. Thus, Beclin1 is able to bind PtdIns3KC3 and additional Atg proteins that organize autophagosome formation. In a normal condition, Bcl2 binds to the complex PtdIns3K-Beclin1; but when Bcl2 is release, the complex PtdIns3K-Beclin1 is activated and the autophagy is prompting by autophagosome formation. Moreover, the protein levels of Beclin1 are controlled by caspase-dependent cleavage, one of the most common signalling cascades involved in apoptosis, and the resulting fragments are unable to induce autophagy [35]. To this end, the apoptosis was studied during ALS progression.

The activated caspases initiate cell death by cleaving several proteins such as PARP-1. Cleavage of PARP-1 by caspases is considered to be a hallmark of apoptosis [43]. However, in our SOD1G93A mice the effectors caspase-3 and caspase-9 as well as PARP-1 cleavage were not detected (Supporting information S1). These results suggested that apoptosis might not be triggered along disease progression in the skeletal muscle from SOD1G93A mice. To verify these results and taking into consideration previous studies *in vitro* in which autophagy protected muscle cells after chemical and physical stimuli[44], we performed a Tunel assay to study more accurately the apoptosis activation. Interestingly and contrary to previous studies that revealed caspase-mediated apoptosis at paralysis stage[45], no significant differences were detected between WT and SOD1G93A mice, reinforcing the fact that apoptosis was not activated in any studied stage of the disease. These results were in accordance with a previous study in a mouse model that lacked the antioxidant enzyme Sod1 (*Sod1* ^*-/-*^ mice). The chronic oxidative stress induced in this animal model exacerbated muscle atrophy, leading to the activation of mitochondrial-mediated myonuclear apoptosis[46]. However, in the skeletal muscle from SOD1G93A mice no apoptosis activation was found, pointing out to the fact that mutant SOD1 was not directly influencing mitochondrial dysfunction as previously reported[30, 39]. Furthermore, in connection with this result it is interesting to note that rapamycin, an autophagy enhancer, accelerated the motor neuron degeneration in SOD1G93A mice and it exacerbated the pathological progression mainly through apoptosis, suggesting that specific stimuli could possibly trigger the tissue degeneration through apoptosis[47].

## Conclusions

The data presented here uncover for the first time that there is a time-point dependent activation of autophagy and UPS in the skeletal muscle from SOD1G93A mice. It has been described their compensatory way of action, in other words, the autophagy activation is prompted when the UPS impairment is present, and therefore autophagy blockage directly influences the UPS function [48]. Based on the fact that a failure of the UPS function is relevant in the neurodegenerative progression in motor neuron diseases, our results are in line with this point, since the activation of proteasome activity was only relevant at early symptomatic stage and this activation was preceded and followed by the autophagy activation at presymptomatic and terminal stages. Taking into consideration that in skeletal muscle, in contrast to other tissues, the activation of autophagy can persist for long periods of time, the transient activation of autophagy at early and late stages in SOD1G93A mice could suggest a deregulation of this process along disease progression due to the degeneration related to ALS. These findings provide new insights into the role of autophagy in the progression of ALS, although further studies are required to completely unravel unknown features of this protein degradation mechanism and its close relation to the UPS.

## Supporting Information

S1 Fig Positive control for Apoptosis. Apoptosis may be induced in experimental systems through chemical agents such as doxorubicin. After confirming that mesenchymal stem cells from SOD1G93A mice were growing by visual inspection, the DNA damaging agent (doxorubicin 2 μM) was added. The cells were checking 24 hours later to determine if cells have begun to apoptose. Finally, the cells were harvested; the protein was extracted and the western blot was performed following the described protocol in Material and Methods. As shown in this figure, PARP-1, caspase-3 and caspase-9 proteins were only detected in positive control (C+). In rest of samples, wild type as well as SOD1G93A mice (at P40, P60, P90 and P120), the cleaved caspase-3, caspase-9 and PARP-1 were no detected.
(TIF)
