# Supplementary figures and images for: Time-Point Dependent Activation of Autophagy and the UPS in SOD1G93A Mice Skeletal Muscle
Source: PLoS One. 2015 Aug 5;10(8):e0134830. doi: 10.1371/journal.pone.0134830 (PMC4526523; doi:10.1371/journal.pone.0134830)

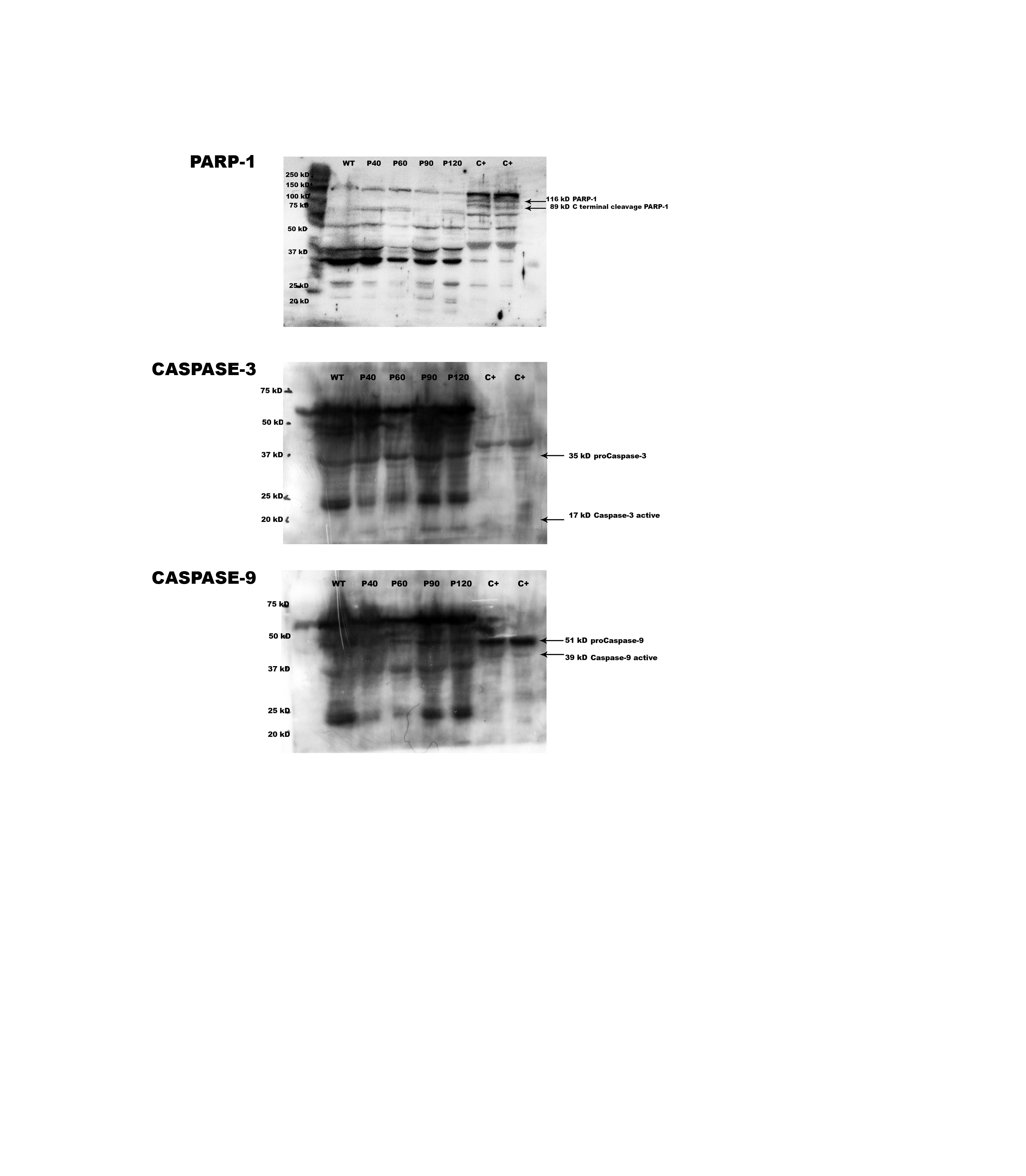

Supplement: S1 Fig — Apoptosis may be induced in experimental systems through chemical agents such as doxorubicin. After confirming that mesenchymal stem cells from SOD1G93A mice were growing by visual inspection, the DNA damaging agent (doxorubicin 2 μM) was added. The cells were checking 24 hours later to determine if cells have begun to apoptose. Finally, the cells were harvested; the protein was extracted and the western blot was performed following the described protocol in Material and Methods. As shown in this figure, PARP-1, caspase-3 and caspase-9 proteins were only detected in positive control (C+). In rest of samples, wild type as well as SOD1G93A mice (at P40, P60, P90 and P120), the cleaved caspase-3, caspase-9 and PARP-1 were no detected. (TIF) [file pone.0134830.s001.tif]
